# A narrative overview of undergraduate geriatric medicine education worldwide

**DOI:** 10.1007/s41999-024-01055-1

**Published:** 2024-09-24

**Authors:** Grace M. E. Pearson, Yoav Ben-Shlomo, Emily J. Henderson

**Affiliations:** 1 Aging and Movement Research Group, Population Health Sciences, University of Bristol Medical School, Canynge Hall, Bristol, BS8 2PN UK; 2 Older Persons Unit, Royal United Hospitals NHS Foundation Trust, Combe Park, Bath, BA1 3NG UK

**Keywords:** Medical education, Geriatric medicine, Ageing, Undergraduate, Medical schools, Medical students

## Abstract

There is a mismatch between the healthcare needs of the ageing population worldwide and the amount of education medical students receive in geriatric medicine. In 2014, Mateos-Nozal et al. published a systematic review of all undergraduate education surveys in geriatric medicine—a decade on, it is timely for an up-to-date overview of the state of undergraduate geriatric medicine education globally. In this review, we outline the international evidence in the field, exploring the results of national and multi-national teaching surveys, and discussing the relative strengths and weaknesses of nationally recommended curricula. We set these findings in the context of ageing population demographics, concluding with recommendations for the future of education and educational research in geriatric medicine, that aims to build capacity in the healthcare workforce and improve quality of care for older people.

## Key summary points

1. The global population over 65 years of age is expected to increase by 120% between 2019 and 2050, yet, by last estimate, up to 27% of the world’s medical schools were not teaching any geriatric medicine.

2. Our aim is to provide an overview of current practice in undergraduate geriatric medicine education globally, serving as an update to the review by Mateos-Nozal et al. (2014).

3. The World Health Organization advocates for national guidelines as a means of increasing and improving educational provision in geriatrics: thus, there exist eight national/multi-national undergraduate curricula, which we have critiqued here.

4. There does seem to be a positive association between population demographics and undergraduate teaching in geriatric medicine; however, we must seek to be more proactive in lower- and middle-income countries where populations are now ageing more rapidly.

## Background

The global population over 65 years of age is expected to increase by 120% between 2019 and 2050, yet, by last estimate, up to 27% of the world’s medical schools were not teaching any geriatric medicine [1, 2]. This presents significant challenges to health and social care systems caring for increasing numbers of older people with highly complex needs, which is at odds with the quantity and quality of geriatric medicine education at medical schools. Undergraduate medical education serves as the foundation of all clinical practice, and therefore, offers a singular opportunity to equip tomorrow’s doctors with the knowledge, skills, and attitudes required to care for our ageing population, regardless of specialty. Our aim is to provide an overview of current practice in undergraduate geriatric medicine education globally, serving as reference for international educators and institutions wishing to improve or implement teaching in ageing and geriatrics.

## How much geriatric medicine is being taught globally to undergraduate medical students?

The World Health Organization (WHO) advocates for the education and training of doctors in ageing and geriatric medicine to prepare healthcare systems and workforces for the ageing population. To this end, they collaborated with the International Federation of Medical Students’ Associations (IFMSA) on two international studies, “Teaching Geriatrics in Medical Education” 1 and 2 (TeGeME) [2, 3]. In TeGeME1, Keller et al. gathered institutional-level data from 161 medical schools in 36 different countries worldwide, half of which were high-income countries and half low- or middle-income. They found that only 25% of schools in high-income countries offered any clinical teaching in geriatrics, which dropped to under 20% in low- and middle-income countries, but didactic teaching was offered in up to 80% of all responding schools. The average duration of any geriatrics teaching was 20–40 hours (h). It remained that 27% of schools surveyed reported no teaching in geriatric medicine (19% in high-income countries). In a more recent systematic review by Mateos-Nozal et al. (2014), the estimated global prevalence of undergraduate education in geriatric medicine was higher, at 81%, however, this varied widely by country (12–100%) and was only mandatory in 62% [4]. Table 1 summarises an update to this 2014 review, detailing the national and regional surveys of undergraduate education in ageing and geriatric medicine to date, which we will explore in more detail.

**Table 1:** An updated summary of national and regional surveys of undergraduate education in geriatric medicine, adapted from Mateos-Nozal et al. (2014) [4]

Country/Region	Area	Publication year	Authors and reference
UK	National	1988	Smith and Williams [26]
Germany	National	1998	Nikolaus [10]
Spain	National	2004	Estrada et al. [14]
Germany	National	2005	Kolb and Vorstand [11]
USA	National	2005	Eleazer et al. [23]
Argentina	National	2006	Bassan et al. [7]
Canada	National	2006	Gordon and Hogan [21]
UK	National	2006	Bartram et al. [27]
USA	National	2007	Warshaw et al. [24]
Spain	National	2008	López Mongil et al. [15]
Europe	Regional	2008	Michel et al. [20]
UK	National	2010	Gordon et al. [28]
Israel	National	2008	Leibovitz et al. [17]
Canada	National	2011	Gordon [22]
USA	National	2012	Bragg et al. [25]
Netherlands	National	2013	Tersmette et al. [13]
Austria and Germany	Binational	2013	Singler et al. [12]
Africa	Regional	2013	Dotchin et al. [5]
UK	National	2014	Gordon et al. [29]
Africa	Regional	2015	Frost et al. [6]
Latin America	Regional	2015	López et al. [8]
Spain	National	2019	Mateos-Nozal et al. [16]
Malaysia	National	2022	Sallehuddin et al. [9]
Poland	National	2024	Kupis et al. [18]

In countries traditionally considered outside of Western and Anglo European culture, where population ageing has hitherto been less marked, provision of undergraduate education in geriatrics is low. For instance, in a 2013 survey of medical education in Africa, only 5 countries (out of 50 with medical schools) reported any teaching in geriatric medicine [5]. In a later study (2015) of 25 medical schools across 11 southern African countries, Frost et al. found that 40% of responding medical schools taught no geriatrics [6]. Of the 60% that taught geriatric medicine, only one was teaching it as an independent specialty, with all others teaching aspects of geriatrics alongside other specialties.

Similarly, in an initial survey of medical students studying in Argentina, only 2% reported having received any teaching in geriatrics [7]. However, a later review of medical education in 308 medical schools across 18 Latin American countries (spanning Central and South America) found that geriatrics was being taught in 107 institutions (35%)[8]. The country with the highest percentage of schools teaching geriatrics was Mexico (82%), however, in most other countries it was less than 50%, and where taught, the majority were teaching geriatric medicine for less than 60 h total. The limited data from Asia was more positive: in a 2019 survey of Malaysian medical schools, amongst the 16 schools that responded 94% covered ageing and geriatric medicine in their undergraduate curricula, with time spent varying widely between 6 h and 6 weeks [9]. 31% (5/16) of responding schools taught geriatric medicine in a stand-alone specialty clerkship, whilst the remainder integrated teaching in geriatrics with other specialties—a necessity, given that only seven of the responding schools had access to geriatric medicine wards.

In Western, Anglo European countries the provision of undergraduate education in geriatric medicine is higher. In Germany, Nikolaus (1998) and Kolb and Vorstand (2005) found that less than half of German medical schools were teaching geriatrics [10, 11]. When considered with neighbouring Austria, this provision had improved to over two-thirds of medical schools by 2013 [12]. By contrast, in the same year, only 25% of medical schools in the Netherlands reported having a clerkship in geriatric medicine, with geriatrics only accounting for 2.2% of the pre-clinical curriculum and 1.5% of questions in their national medical student assessment, the cross-institutional progress test [13]. A comparison of healthcare profession degrees in Spain found that an average medical degree included 127.5 credits in subjects related to gerontology and geriatrics, which the authors claim is equivalent to 1275 h of teaching time—still less than nursing and psychology majors—but less than half of these were mandatory or core subjects [14]. In 2008, a survey of 29 medical schools in Spain found that 72% were teaching geriatrics, but only 52% on a mandatory basis, and 55% as an independent specialty [15]. By 2015, this had increased marginally to 78%, with a mean teaching duration of just 36 h [16]. In Israel, there is coverage of geriatrics in all four medical schools, with a nationally mandated 2-week clinical clerkship [17]. More recently (2024), a survey by Kupis et al. found that 95% (18/19) of Polish medical schools offered teaching in geriatric medicine, but this was very heterogeneous (range 11–60 contact hours, majority lecture-based), and still only 2.9% of learning objectives (LO) in the national educational standards were specifically related to geriatrics [18].

Mateos-Nozal et al. cite three multinational European surveys of undergraduate education in geriatrics [4]. We were unable to obtain the full-text for the first of these,^1^ and found that the second was not a survey of undergraduate teaching [19]. This leaves just one European-wide survey by Michel et al. (2008), who found that geriatrics was taught in 25/31 (81%) of the European countries surveyed [20]. However, geriatrics was taught to “widely differing extents”, for example teaching time varied from 100 h in Norway to < 10 h in Ireland, Luxemburg, and Turkey. Moreover, only 16 countries (60%) had organised clinical clerkships in geriatric medicine, which were mandatory in just 11.

Our searches identified five surveys from North America: three of the United States of America (USA), and two from Canada. In 2006, Gordon & Hogan found that only 50% of Canadian medical schools had a mandatory clerkship in geriatric medicine, with an average of 78 h teaching [21]. When resurveyed in 2011, 100% then had geriatrics content in pre-clinical teaching, with a mean of 21 h [22]. However, the prevalence of mandatory clinical rotations in geriatric medicine had decreased slightly to 44%, but the mean duration of clinical teaching had increased to 82 h.

By contrast, between 1999 and 2000, Eleazer et al. found that 89% of responding US medical schools (both osteopathic and allopathic)^2^ included care of older adults in their undergraduate curricula [23]. Only 64%, however, had defined LOs in geriatrics, and this only translated to an average of 14.4 h didactic teaching (clinical teaching time was not reported). More descriptive data was collected in subsequent surveys: in a 2005 survey with a 68% response rate, 23% of responding schools had a mandatory clerkship dedicated to geriatric medicine, and a further 48% had integrated geriatrics into other clinical rotations [24]. Concerningly, however, 17% of reporting schools were relying on clinical exposure to older patients without a structured curriculum or LOs. By 2010, the prevalence of mandatory geriatrics clerkships amongst reporting schools had improved to 27%, and elective clerkships to 87%—both generally of 4 weeks duration [25].

Although the UK did not participate in TeGeME1 [2], a resurvey by WHO/IFMSA in 2007 (TeGeME2) estimated that 15% of UK institutions were not delivering any teaching on geriatric medicine [3].^3^ This estimate is a reduction when compared to a prior study of teaching provision in the UK (1988), which found that 25/27 (93%) schools included geriatrics in their undergraduate medical training [26]. However, there was a documented decline in the UK, where decades later (2006), a survey found that 21/30 (70%) of responding medical schools in the UK were teaching geriatrics, and only two of these taught it as an independent specialty [27]. These proportions have only deteriorated further with time: in 2008, 17/30 (57%) schools were teaching geriatrics, and in 2013, 19/30 were (63%) [28, 29]. In 2008, the mean time spent on geriatrics was just 49 h, which had increased to an average of 55.5 h by 2013, but this still equated to less than 2 weeks’ teaching time.

## How is geriatric medicine being taught at medical schools globally?

Many of the surveys outlined above identified that the quantity and quality of undergraduate education in geriatric medicine was closely linked to the presence of institutional research in geriatrics, or geriatricians in the medical school faculty. Thus, in their TeGeME studies, the WHO/IFMSA strongly weighted university faculty presence in their assessment of teaching quality, termed the GERIND score (Table 2), and the content and methods of teaching formed a smaller part of their evaluation [2]. In their assessment, Norway achieved the highest GERIND score of 95.4/100, and the State of Palestine the lowest, just 4.0/100. Many of the surveys described above include details of the ‘hows’, ‘whens’, ‘wheres’ and ‘who bys’ of undergraduate education in geriatrics lacking from TeGeME. In general, teaching is multimodal and delivered by multidisciplinary teams—corroborated by a recent review by Masud et al. [30].

**Table 2:** GERIND score created for the World Health Organization (WHO) Teaching Geriatrics in Medical Education (TeGeME) study

Facilities for clinical training in geriatric medicine: Quantitative criteria (up to 60 points)	
Independent university faculty unit and university hospital ward	60 points
Independent university faculty unit or university hospital ward	30 points
University faculty sub-unit and university hospital sub-ward	40 points
University faculty sub-unit or university hospital sub-ward	20 points
Independent university faculty unit and university hospital sub-ward	50 points
Independent university hospital ward and university faculty sub-unit	50 points
Other form of teaching (yes)	10 points
Intention to include in the future (yes)	10 points

Non-clinical, didactic teaching in geriatric medicine: qualitative criteria (up to 40 points)	
Contents of taught elements	Up to 20 points
Mandatory (yes)	15 points
Coverage of life course perspective (yes)	5 points

This was used to compare undergraduate education in ageing and geriatric medicine between medical schools and between nations. Each country was given a national average GERIND score, whilst each medical school’s score was weighted by the number of students. Adapted from Keller et al. (2002) [2]

Masud et al. also found that teaching in geriatric medicine is most often delivered in a vertical, or longitudinal fashion at multiple points in undergraduate curricula, rather than in single units or clerkships [30]. There are many advantages to this approach: vertical integration means that learning of ageing and the complexities of geriatric medicine can be reinforced through repeated exposure and is more reflective of clinical reality [31]. Moreover, lots of these broad concepts core to geriatric medicine are applicable to multiple specialties, and joining forces in this manner alleviates pressure in time- and resource-restrained curricula. However, learning in geriatric medicine may be prone to ‘erosion’ or ‘dilution’ if there is no dedicated time and teaching [32]—for “time spent around older patients is not the same as time spent learning about frailty, complexity and cognitive impairment”[29]. Indeed, it is demonstrable that medical students can learn these concepts most effectively on dedicated geriatric medicine clerkships [33, 34]. This maybe because it is more difficult for students and educators to identify LOs when they are integrated longitudinally. This is demonstrated by Nagoshi et al., who in their evaluation of a longitudinal, integrated curriculum in geriatrics, found that whilst skills improved, attitudes were static and knowledge worsened [35].

## What national recommendations exist globally to guide undergraduate geriatric medicine education?

In TeGeME1, Keller et al. received national-level data from 72 countries, of which just 53% had national regulations for undergraduate medical education [2]. Of this 53%, only 41% of national standards or curricula mentioned geriatric medicine. Therefore, in their conclusion to TeGeME, the WHO advocated for national guidelines as a means of increasing and improving educational provision in geriatrics [2]. In 2006, the International Association of Gerontology and Geriatrics (IAGG) published a guide comprising 15 core learning objectives for medical students in geriatrics, however, this is no longer available [36, 37]. There still exist several national and international undergraduate curricula written by expert consensus, delineating minimum requirements for medical graduates in the care of older adults [30]. Generally, these national curricula were developed using consensus methods, usually a modified Delphi technique beginning with a literature review and curricular mapping, followed by a consensus conference or panel [38–42].

On review of published curricula, we found that the USA appears to be the first country to have implemented national standards in this field, opting for a competency-based approach (summarised in Table 3) [38, 43, 44]. The iterative improvement of these American guidelines is evident, progressing from a basic ‘knowledge, attitudes, skills’ framework to Tinetti’s ‘5 M’ model for geriatric care [45], thus making the undergraduate competencies directly translatable to, and implementable within existing clinical practice. The Canadian Geriatrics Society undergraduate competencies are divided into nine domains, very similar to the 2009 iteration of the USA curriculum by Leipzig et al., except for they are primarily skills and knowledge focussed, with no reference to attitudes [46]. There is also a “minimum contents for medical school programs in geriatric medicine” for Latin America, comprising 15 topic areas with proposed departments to teach each topic and suggested teaching duration [47].

**Table 3:** A summary of updates to the American competencies in geriatric medicine for undergraduate medical students. The numbers in brakets refer to the number of learning objectives (LOs) associated with each area

	First	Second	Third
Authors	Eleazer et al. [43]	Leipzig et al. [38]	Lundebjerg et al. [44]
Year	2000	2009	2021
Contents	23 competencies covering the “basic attitudes, knowledge, and skills necessary for the care of older patients” Attitudes (7) Knowledge—basic science (7), clinical (7) Skills—assessment (1), diagnosis (1)	26 “minimum geriatric competencies for medical students” in 8 domains Medication management (3) Cognitive and behavioural disorders (5) Self-care capacity (3) Falls, balance, and gait disorders (2) Health care planning and promotion (3) Atypical presentation of disease (2) Palliative care (3) Hospital care for elders (5)	27 “minimum geriatric competencies for medical students” in 5 domains (Tinetti’s 5Ms) [45]: Mind (4) Mobility (3) Medications (4) Multicomplexity (11) Matters most (5)

Faced with a similarly rapidly ageing population to Anglo European countries, Japan also implemented a national undergraduate curriculum in geriatric medicine early-on: the first Japanese “model core curriculum” in geriatric medicine for undergraduates was published in 2001, consisting of 18 LOs [48]. This was adapted in 2010 into 7 domains and updated once again in 2016 into 2 broad categories of “classroom education” (26 LOs) and “practical education” (10 LOs) [48, 49]. More recently (2020), Sallehuddin et al. published a Malaysian undergraduate geriatric medicine curriculum of 34 LOs in 10 domains [39].

There are three multi-national recommended undergraduate curricula in geriatrics: Latin America (detailed above), Australasia, and Europe [40, 47, 50]. Similar to the original USA recommended curriculum by Eleazer et al., the Australian and New Zealand Society for Geriatric Medicine “Position Statement on Education in Geriatric Medicine”, first published in 2006 and updated in 2021, divides recommended LOs into “essential areas of knowledge” (18 LOs), “essential skills” (8 LOs), and “essential attitudes” (5 LOs) [43, 50]. What sets this curriculum apart, however, is the extensive advice given on identifying and effecting learning opportunities to implement the curriculum, divided into ‘how’, ‘where’, ‘when’, and ‘by whom’ geriatrics teaching should be delivered. Conversely, the European curriculum adopts a similar structure to the second iteration of the USA curriculum, with 10 broad domains, each with 2–6 LOs clustered beneath them. This European curriculum has the widest remit and contributorship (29 countries), since the IAGG guidelines are no longer available [38, 40].

There also exist several recommended undergraduate curricula for specific conditions and sub-specialties related to geriatric medicine, namely: older age psychiatry and pharmacology [41, 42, 51]. Similar to the broader geriatrics curricula written by Eleazer et al. and Graham et al. [43, 50], the American “geriatric mental health learning objectives for medical students” and the international “geriatric clinical pharmacology curriculum for medical students” are divided into knowledge, skills, and attitudes and then classified into six and eight domains, respectively [42, 51]. A specific curriculum for delirium was written by consensus at the European Delirium Association Conference in 2015, comprising three sections: (1) aetiology, epidemiology, and pathophysiology, (2) diagnostics, and (3) management [41]. Like the Australasian guidance, this curriculum presents specific suggestions for ‘how’, ‘by whom’, ‘when’ and ‘where’ delirium should be taught to undergraduate medical students.

After successive national surveys of medical school curricula in the UK found that there had been a decline in the provision of teaching on geriatrics, the British Geriatrics Society (BGS) outlined four mechanisms by which this could be improved, namely: a national curriculum, development of academic geriatrics, grant-funding, and teaching innovation [36]. In similar fashion to the conclusions drawn by the WHO from TeGeME, the BGS recognised the importance of national curricula in assuring the quantity and quality of medical education in geriatrics. Thus the BGS recommended undergraduate curriculum in ageing and geriatric medicine was first written based on expert consensus in 2008, and subsequently updated in 2013 based upon reviews of national guidance [52]. The 2013 BGS curriculum was comprehensive in its coverage of geriatric medicine and undergraduate competencies (*i*.*e*. knowledge, skills, and attitudes), however, it lacked cohesive structure like other national curricula—LOs were simply numbered, not grouped under sense-making headings. Hence, in 2023 the BGS recommended undergraduate curriculum was updated using a two-stage process of curriculum mapping and nominal group technique (a form a consensus conference) [53]. The curriculum was restructured into seven sections, each with one or two overarching LOs considered core to geriatric medicine, which are expanded and clarified in further sub-LOs, giving structure lacking from previous iterations. This represents the most recent national-level guidance for undergraduate education in geriatric medicine. It remains though, that whilst the updated curriculum is aligned with UK regulatory requirements, its implementation, and that of other similar curricula [39, 40], could be further encouraged through inclusion of specific guidance for educators and institutions, as in the Australasian and delirium curricula [41, 50].

## Conclusion

It is clear from this overview that globally, undergraduate geriatric medicine education is currently falling short in many areas. As demonstrated by Table 4, there does seem to be a positive association between population demographics and undergraduate teaching in geriatric medicine, however, we must seek to be more proactive in lower- and middle-income countries where populations are now ageing more rapidly. As part of this, repeat national and international surveys of teaching provision are timely, given that many of the articles cited here were published over a decade ago, and there is still lots of missing data (Table 4). Equally, perhaps the focus in countries where geriatric medicine is better established can now turn to ‘clarificatory’ research, seeking to improve understanding of the ‘how’, ‘by whom’, ‘when’, and ‘where’ of best practice in undergraduate geriatric medicine education [54].

**Table 4:** Summary of the provision of undergraduate education in geriatric medicine (or national/multi-national curriculum guidance) in comparison with population demographics

Area	Average life expectancy (years), both sexes in 2019^a^		Average percentage total population aged over 65 in 2019^b^	Undergraduate coverage of geriatric medicine by last estimate	(Multi-)National undergraduate curricula in geriatrics
	At birth	At 60			
Africa	65	17	4%	10–11%	No
Australia	69	18	6%	No multi-national data	Australia/New Zealand
Asia	74	20	7%	No multi-national data	Japan; Malaysia
North & South Americas	75 (79 USA; 82 Canada)	21 (23 USA; 25 Canada)	9% (16% USA; 18% Canada)	USA 69% (16% mandatory)	USA; Canada; ‘Latin American’
				Canada 100% (44% clinical)	
				‘Latin America’ (17 countries) 35%	
Europe	79	22	19%	‘Europe-wide’ (33 countries) 81%	UK; European

^a^Average life expectancy data derived from the WHO Global Health Observatory [55]. It should be noted that whilst the average life expectancy at birth for African countries is 65, the five countries worldwide with the lowest life expectancy at birth (51–58) are all African countries (Lesotho, Central African Republic, Somalia, Eswatini, and Mozambique). Conversely, globally the five countries with the highest life expectancy at birth (83–84) are in Asia (Singapore, South Korea, and Japan) and Europe (Spain and Switzerland)

^b^Average percentage total population aged over 65 years derived from the World Data Bank [56]

Understanding that evidence drives policy, we hope that this updated overview will push meaningful change in culture and practice, which would see the mandatory inclusion of geriatric medicine in medical school curricula in a manner and duration proportionate to changing population demographics, and reflective of the diversity of ageing and geriatric medicine. We have highlighted here several evidence-based nationally recommended curricula, which can serve as the base from which to enhance the representation of geriatricians in university faculties, longitudinally weave complex concepts around ageing throughout medical curricula, and most importantly, implement dedicated clinical attachments in geriatrics, all with a view to, ultimately, optimizing the quality of care for older people.

## Data Availability

As this is a narrative overview, please refer to the reference list for original source material.

## Footnotes

1 The University of Bristol and the Royal United Hospital Bath libraries do not subscribe to the relevant journal, and this article was not available in print or electronically for inter-library loan in either institution.

2 Allopathic medicine is the classical form of medicine, as we think of it in Europe. The curriculum for osteopathic medicine is virtually indistinguishable, except for the underlying osteopathic philosophy. This philosophy is that “structure determines function” and that all systems of the body are interconnected, therefore the osteopathic approach focusses on holism and includes training in musculoskeletal manipulation. Graduates from osteopathic medical schools use the postnominals DO, and those from allopathic medical schools use MD. Training is generally of the same length and subject to the same licensing requirements and regulations [57].

3 This report is no longer available online.
